# Preventing an 8-mm Port Site Hernia in Robot-Assisted Laparoscopic Surgery: Insights From Two Rare Cases and Future Preventive Measures

**DOI:** 10.7759/cureus.56609

**Published:** 2024-03-20

**Authors:** Hirotaka Seike, Keiji Nagata, Ippei Yamana, Takahisa Fujikawa

**Affiliations:** 1 Surgery, Kokura Memorial Hospital, Kitakyushu, JPN

**Keywords:** postoperative complication, intestinal obstruction, 8-mm port site incisional hernia, robot-assisted laparoscopic surgery, robotic surgery

## Abstract

Port-site incisional hernia (PIH) is an uncommon complication that can arise subsequent to a laparoscopic procedure, potentially leading to severe adverse effects such as intestinal obstruction. We currently present two cases of incarcerated hernia that occurred at an 8-mm trocar site after robot-assisted laparoscopic surgery (RALS). While occurrences of an 8-mm port-site incisional hernia are infrequent, it is imperative to note that most PIH cases are due to inadequate fascial closure of the port site. Therefore, surgeons must pay attention to closing the fascia of an 8-mm trocar site following RALS.

## Introduction

Robot-assisted laparoscopic surgery (RALS) has several advantages over conventional laparotomy, including less blood loss, detailed observation of tissue and layered structures through the use of a stable 3-D endoscope, and more detailed and precise movements than laparoscopic forceps. As a result, there has been a rapid increase in several facilities introducing robots and performing RALS in recent years [1,2]. On the other hand, laparoscopic surgery, including RALS, has unique complications that are rare in open surgery.

Port-site incisional hernia (PIH) is a rare complication following a laparoscopic procedure, in which the incidence is reported to be 0.5-5% [3-6], and it may cause serious adverse outcomes such as incarcerated hernia [3,7]. In particular, it has been reported that obese patients are at higher risk of developing PIH as they tend to have increased intra-abdominal pressure [8].

The closure of port sites larger than 10 mm is a common practice because most reports of PIH occurred at port sites larger than 10 mm [4] while there is limited consensus on the necessity of closing the 8-mm trocar sites following RALS. This article describes two cases of PIH that developed at an 8-mm trocar insertion site after RALS. In this report, we describe the possible causes of PIH, especially 8-mm PIH, and preventive measures.

## Case presentation

Case 1

**Figure 1:**
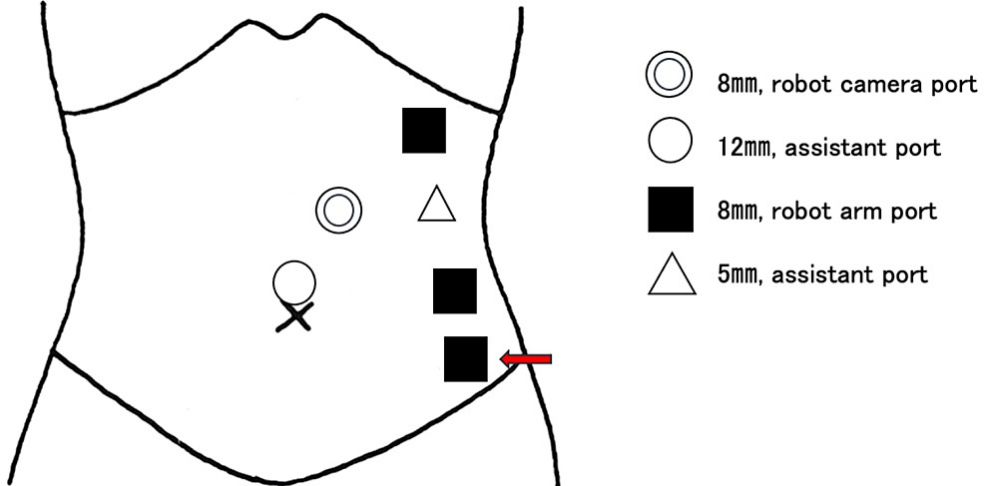
Trocar placement for robot-assisted left nephrectomy in Case 1 A double circle represents an 8-mm robot camera port. The circle represents a 12-mm port, and the triangle represents a 5-mm assistant port. Squares represent an 8-mm robot arm port. The red arrow indicates the location of the port-site incisional hernia (the figure is the authors’ creation).

An 84-year-old Japanese woman was diagnosed with left renal cancer (cT1N0M0) and underwent a robot-assisted left nephrectomy. She had no previous history of laparotomy. Her height, body weight, and body mass index (BMI) were 148 cm, 45 kg, and 20.5 kg/m^2^, respectively. During the surgery, the port for the robot camera was originally positioned above the navel, and the remaining ports were placed according to the instructions provided in Figure 1.

**Figure 2:**
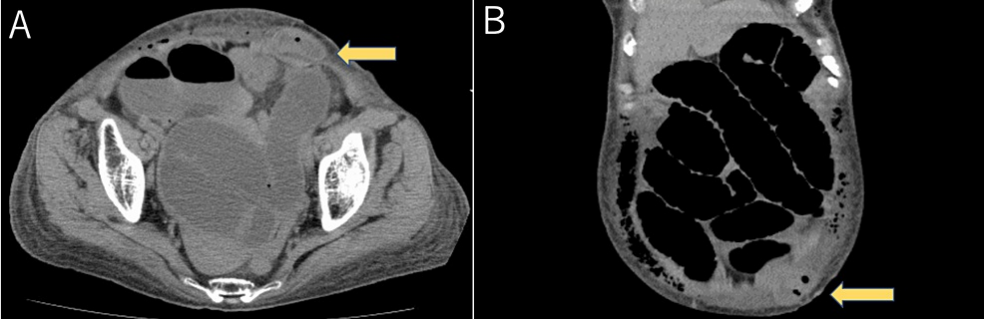
Postoperative axial (A) and coronal (B) CT images in Case 1 The images showed the small intestinal incarceration through an anterior abdominal wall (yellow arrow) with surrounding subcutaneous edema.

The overall operative time and console time were 402 and 316 minutes, respectively. The blood loss was 52 mL. The intraabdominal pressure was maintained at 10 mmHg. Although the access port site of the AirSeal System (SurgiQuest Inc., Milford, CT, USA) was closed using an absorbable suture, no fascia sutures were performed at the sites of the 8-mm ports. There were no specific issues during the initial postoperative procedure. Nevertheless, abdominal pain and vomiting manifested on postoperative day (POD) 4. Despite the insertion of a gastric tube, the symptoms did not improve. On POD 6, the CT scan clearly showed a herniation of the small intestine through an 8-mm trocar site. (Figure 2).

**Figure 3:**
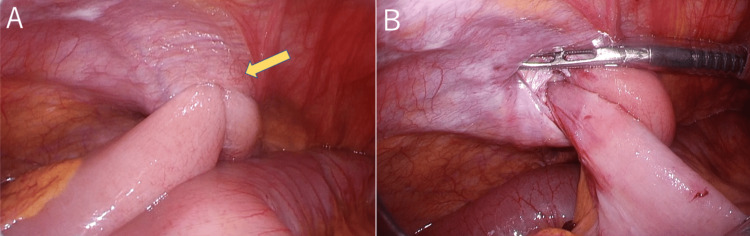
Laparoscopic findings of the small intestine incarcerated at the 8-mm port site. (A) The yellow arrow showed the incarcerated small intestine. (B) The incarceration of the small intestine was successfully removed using forceps.

An emergency exploratory laparotomy was performed due to the incarceration of the small bowel. We initially positioned a 12-mm port above the navel for the laparoscopic camera, followed by the addition of two 5-mm ports on the right side of the abdomen. The laparoscopic examination confirmed that the small intestine had partially prolapsed and become trapped between the peritoneum and transversus abdominis fascia at the previous 8-mm trocar site. We successfully removed the incarceration laparoscopically using forceps (Figure 3).

The small intestine was not necrotic, so intestinal resection and reconstruction were not performed. The fascia and peritoneum of the port site were closed confidently utilizing the laparoscopic extra-abdominal suturing technique via the port closure needle (Endo Close™, Medtronic, Minneapolis, MN, USA). The patient was discharged seven days after the reoperation, and her recovery went well during the follow-up.

Case 2

**Figure 4:**
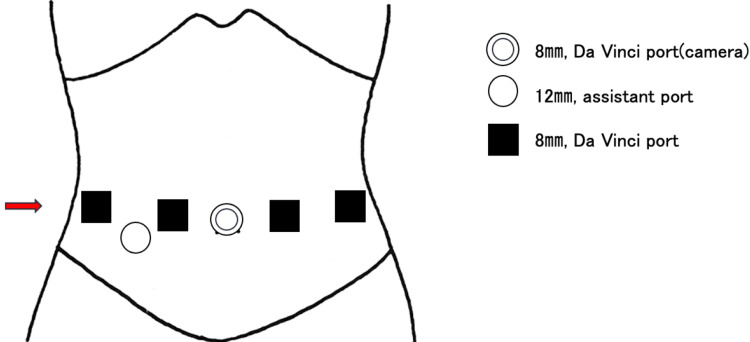
Trocar placement for robot-assisted distal gastrectomy in Case 2 The double circle represents an 8-mm robot camera port. The circle represents a 12-mm assistant port. Squares represent an 8-mm robot arm port. The red arrow indicates the location of the port-site incisional hernia (the figure is the authors’ creation).

Another case was an 86-year-old Japanese woman who was diagnosed with gastric cancer (pT1bN1bM0 stage IIA) and underwent robot-assisted distal gastrectomy (RDG). Her height, body weight, and BMI were 145 cm, 42 kg, and 19.9 kg/m^2^, respectively. She had no previous history of laparotomy. During the surgery, the first port was positioned for the robot camera above the navel, and the remaining ports were placed according to the instructions provided in Figure 4.

**Figure 5:**
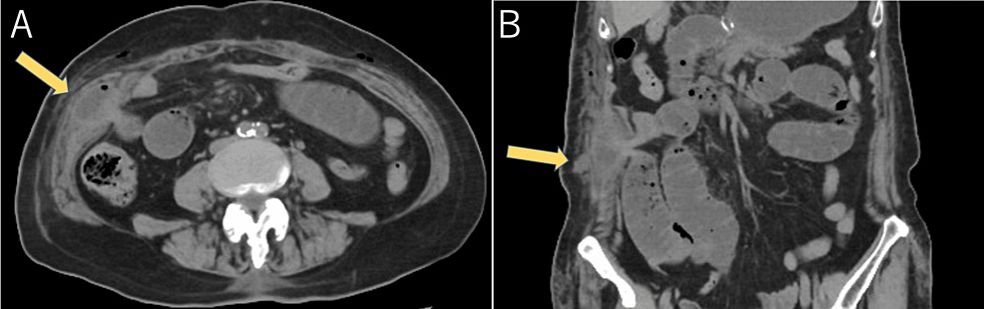
Postoperative axial (A) and coronal (B) CT images in Case 2 The images showed the small intestinal incarceration in the right abdomen (yellow arrows).

The overall operative and console times were 421 minutes and 356 minutes, respectively. The blood loss was 20 mL. Intraabdominal pressure was maintained at 10 mmHg. We used an absorbable suture to close the access port of the AirSeal System, although the fascia of the 8-mm port sites were not closed. There were no specific issues during the initial postoperative procedure. However, she expressed strong abdominal pain and felt sick on POD 9. An abdominal CT scan showed that the small intestine was trapped at the 8-mm trocar site, which was for the arm port of the RDG (Figure 5).

We performed an emergency explorative laparotomy immediately. At the lateral 8-mm port site, we found incarceration of the small intestine. We incised the fascia and released the strangulation of the small intestine under direct vision because it was incarcerated tightly. Although the small intestine was strangulated, we did not perform an intestinal resection because the incarcerated small intestine was not necrotic. We closed the fascia and peritoneum of the hernia orifice using polydioxanone sutures (PDS® Plus Antibacterial Suture, Ethicon Inc., Somerville, NJ). After the reoperation, the patient was discharged from the hospital 12 days later, and her recovery went well during the follow-up.

**Figure 6:**
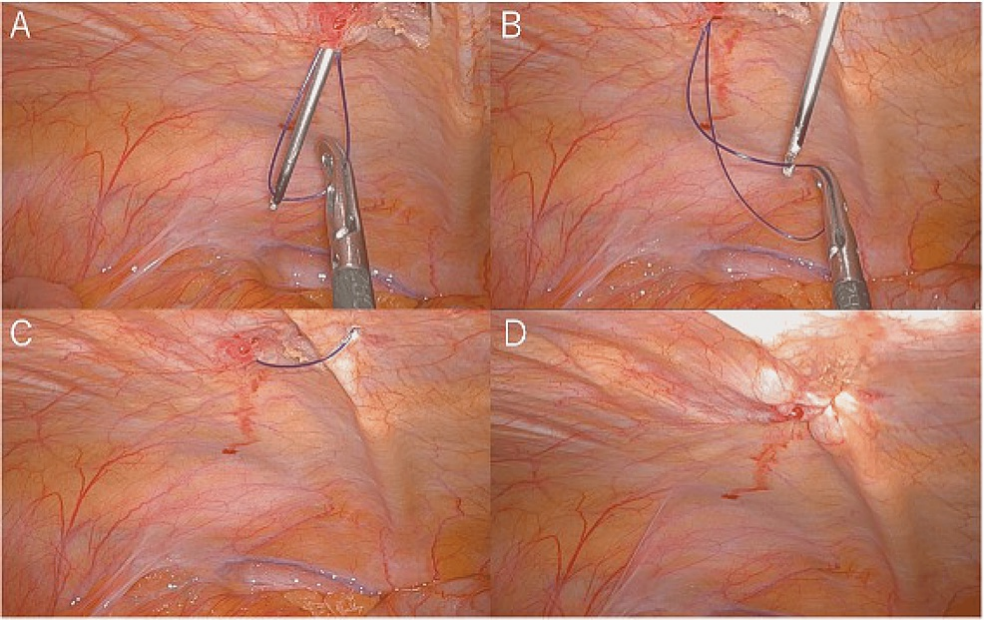
The laparoscopic extraperitoneal suture approach. (A) The suture is grasped at the tip of the Endo Close™ and inserted through the abdominal wall into the abdominal cavity, and this suture is passed to the forceps inserted through the other port. (B) Endo Close™ is inserted from the opposite side of the port hole, and the suture grasped by the forceps is passed to the Endo Close™. (C) The Endo Close™ is pulled out of the body. (D) The muscle layer and peritoneum are stitched together by ligating and closing the stitches outside the body.

Since these two cases, we currently use the laparoscopic extraperitoneal suture approach to cover all of the fascia at the 8-mm trocar sites. The closing method is shown in Figure 6.

## Discussion

PIH is an uncommon complication that can arise subsequent to a laparoscopic procedure, including RALS, potentially leading to severe adverse effects such as intestinal obstructions [3,7]. In the literature, Boughey et al. reported that the incidence of PIH is less than three per 1,000 cases [9]. In another report, Montz et al. reported that the occurrence of PIH following laparoscopic surgery has been calculated at 21 per 100,000 laparoscopic surgeries [3].

There have been reports of risk factors associated with an increased likelihood of developing PIH. The patient’s risk factors include age (60 years or older), obesity (BMI of 28 kg/m^2^ or more), diabetes, and the presence of wound infection [6]. The operation’s risk factors include trocar type, port diameter, insertion position, the presence or absence of fascial closure, thread type, etc. [4,10]. It has been reported that obese patients have an increased risk of developing PIH because of a tendency toward increased intra-abdominal pressure [8]. On the other hand, there are reports that the risk of developing port-site hernia does not change with a higher BMI [11]. In our two cases, the patient had no risk factors leading to PIH, including obesity, diabetes, wound infection, or the sharp tip of the trocar-obturator, except for age and lateral port position.

The incidence of PIH following a laparoscopic procedure depends on the trocar size. It has been reported that most PIH cases were associated with trocars larger than 10 mm in diameter while only 2.7% occurred with the use of trocars less than 8 mm in diameter [12]. We routinely close the fascia of 12-mm trocar sites due to the higher risk of PIH. However, there is debate on whether we should routinely close the fascia of an 8-mm trocar site.

Ogasa et al. focused on the fact that in RALS, the port moves conically around the remote center and demonstrated that the diameter where the port passes through the peritoneum, which is far from the remote center, is wider than the port diameter [13]. They pointed out that the abdominal wall defect may widen depending on the location of the remote center.

Robotic arms are typically positioned more to the side than laparoscopic ports to adhere to the 10-cm rule, which prevents collisions between the arms. This is especially true for thin patients. Therefore, the robotic trocars are positioned where the abdominal fascia weakens [14]. In the current two cases, both patients were thin rather than obese, and the PIH occurred in the lateral abdomen, where the abdominal fascia weakens. This may have been one of the possible causes of the 8-mm PIH.

Complete and secure sutures, including the peritoneum, are necessary for closing the trocar site. However, it is difficult to directly view the fascia and peritoneum at an 8-mm port, especially in cases with thick subcutaneous obesity. In such cases, it is sometimes difficult to suture the fascia from outside the body, so a direct visual closure of the fascia and peritoneum with the use of a laparoscope is also effective [15]. Since these two cases, we currently close all the fascia of 8-mm trocar sites with the laparoscopic extraperitoneal suture technique via a port closure needle (Endo Close™). No port-site hernia was observed in subsequent cases. PIH at an 8-mm port site following RALS has been reported in recent years; however, more research would be needed to establish the cause of this complication of 8-mm port site hernia and the indication for fascial closure of trocar sites after RALS.

## Conclusions

We experienced two cases of 8-mm PIH with an acute small intestinal obstruction after RALS. The robotic trocar is inserted more laterally, where the abdominal fascia weakens, especially in thin patients, and PIH can occur even in thin patients, as in the current cases. To prevent port site herniation, it is preferable to close all fascia at the 8-mm trocar site laparoscopically under direct vision after RALS.
